# Digital Workflow for a Two-Piece Hollow Bulb Obturator in Maxillary Defect Rehabilitation: A Clinical Case Report

**DOI:** 10.1155/crid/1058055

**Published:** 2025-11-11

**Authors:** Isabel Gomes, João Paulo Martins, Cátia Branco, Luís Miguel Lopes

**Affiliations:** ^1^Oral and Biomedical Sciences and Research Unit (UICOB), Faculty of Dental Medicine, University of Lisbon, Lisbon, Portugal; ^2^Faculty of Dental Medicine, University of Lisbon, Lisbon, Portugal; ^3^Laboratory for Instrumentation, Biomedical Engineering and Radiation Physics (LIBPhys), Lisbon, Portugal

**Keywords:** definitive obturator, maxillectomy, palatal defect

## Abstract

Maxillectomy defects after oncologic surgery can create a communication between the nasal and oral cavities, leading to significant challenges in mastication, swallowing, speech, and facial aesthetics. Prosthodontists play a critical role in rehabilitating such defects through obturator prostheses. This case report presents a digital workflow for fabricating a two-piece hollow bulb maxillary obturator for a patient with a large acquired maxillary defect and severe trismus. The prosthesis comprises a hollow bulb component and a denture segment, which interlock using neodymium magnets. The two-piece obturator was digitally planned and 3D-printed, resulting in a lightweight, aesthetically pleasing, and easily insertable prosthesis. This approach effectively improved the patient's quality of life, demonstrating the advantages of digital design in complex prosthodontic rehabilitation.

## 1. Introduction

The global incidence of cancer has been progressively rising, predominantly because of demographic expansion, population aging, and the escalating prevalence of modifiable risk factors, including obesity, tobacco consumption, and inadequate dietary patterns [1, 2]. In Portugal, approximately 1500 new cases of oral cancer are diagnosed each year, with around 1250 occurring in men and 250 in women [3]. The main risk factors associated with oral cancer include tobacco use, alcohol consumption, and infection with human papillomavirus (HPV), particularly in oropharyngeal cancers [4–6].

Malignant tumors of the maxilla are typically classified according to their tissue of origin, including squamous cell carcinomas, tumors of the salivary glands, mesenchymal neoplasms, and other less common malignancies [2, 4, 7]. Treatment of these tumors includes surgical removal with safety margins, followed by radiotherapy sessions [8]. Maxillary defects following surgical resection often give rise to a range of functional and aesthetic complications. These may include hypernasal speech, nasal fluid leakage, increased risk of aspiration, facial disfigurement, and reduced masticatory efficiency [9]. Therefore, treatment of maxillary defects through reconstructive surgery and/or prosthodontics is crucial for these patients' recovery.

Bone/skin grafts and advances in microvascular surgery have allowed many oncology patients with palatal tumors to have these resected and immediately reconstructed after the surgery [10]. However, reconstruction of the resulting defect is not always possible, and in many cases, patients are left with defects and need prosthetic rehabilitation to resolve posttreatment difficulties. If the resection site cannot be closed surgically, an obturator is required to restore the separation between the nasal and oral cavities. The obturator will improve chewing, swallowing, speech, dental aesthetics, facial support, and overall quality of life [10, 11].

Patients who have had a maxillectomy without surgical reconstruction typically undergo several stages of prosthetic treatment. Initially, a surgical obturator is made and worn for 1–4 weeks immediately after the procedure. Then, an interim obturator is constructed and worn for 3–6 months until the defect heals. Finally, when the defect achieves stability in terms of shape and size, a long-term obturator is made [11].

The effective seal of the defect is crucial to improve all the affected system functions [10], and various types of obturators have been used to achieve that, such as hollow bulbs (open and closed), full bulbs, and two-piece obturators [2]. Obturators with a hollow design are often preferred for their lightweight and comfort [12, 13]. Furthermore, gravity is a dislodging factor affecting prosthesis stability and retention and is especially significant in patients with large defects and heavy obturators [12].

Whenever feasible, obturators are fabricated as single-piece prostheses. However, when the size of the defect to be sealed demands a prosthesis with dimensions that prevent intraoral insertion, a two-piece design is considered. Moreover, in some cases, the shape and insertion path of the obturator may be incompatible with the patient's limited mouth opening [14]. Trismus is a common complication in patients who have undergone maxillectomy followed by radiotherapy. Fibrosis of the masseter and lateral pterygoid muscles can lead to a marked restriction in mouth opening, making prosthodontic procedures challenging, both for the clinician during impression taking and for the patient when attempting to insert the prosthesis [15].

This case report is aimed at describing a clinical and laboratory workflow for fabricating a two-piece obturator with a closed hollow bulb, utilizing an optical 3D scanner, computer-aided design (CAD) software, and rapid prototyping technology (3D printing).

## 2. Case Report

A 64-year-old male patient was referred to the Department of Removable Prosthodontics at the Faculty of Dental Medicine, University of Lisbon, with a chief complaint about a previously fabricated obturator.

During the anamnesis, the patient stated that, in August 2018, he underwent a biopsy due to a painful swelling in his right hard palate and was diagnosed with squamous cell carcinoma of the right maxillary sinus (T4N0M0). In January 2019, he underwent a right maxillectomy for surgical removal of the carcinoma, without reconstruction of the defect. Postoperatively, the patient received chemotherapy (cisplatin) and radiotherapy, which concluded in April 2019. The oncologic treatment resulted in oronasal communication due to a large palatal defect, along with severe trismus, cataract in the right eye, and hearing loss on the same side.

**Figure 1:**
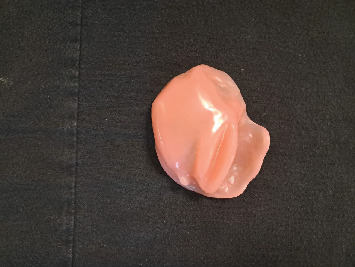
Obturator without teeth used by the patient (intaglio surface).

**Figure 2:**
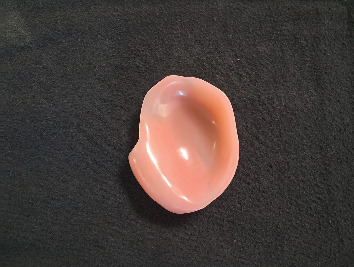
Polished surface of a toothless obturator.

Due to the trismus and associated challenges, the patient had been using single-piece obturators without teeth (Figures 1 and 2). However, he complained of poor aesthetics (no upper teeth), inadequate retention, food accumulation beneath the obturator, and impaired phonetics, with speech often becoming unintelligible—factors that significantly affected his daily life. As a result, the patient expressed a desire to replace the obturator with a more suitable alternative, preferably including teeth.

**Figure 3:**
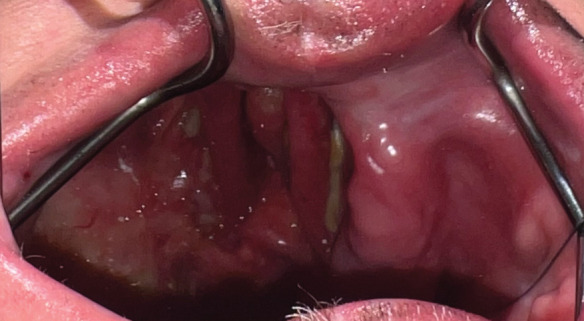
Intraoral photograph showing the extent of the defect and the remaining edentulous ridge.

**Figure 4:**
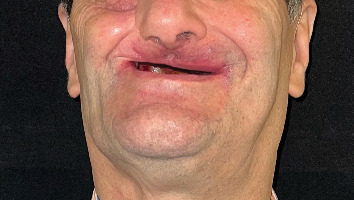
Frontal view of the patient with the previous obturator in place, illustrating perioral skin changes and diminished vertical height of the lower third of the face.

Intraoral examination revealed a defect on the right side extending across the midline, classified as Class IV according to Aramany's classification of maxillary defects (Figure 3) [16]. The remaining left maxillary arch was edentulous, while the mandibular arch was fully dentate. Extraoral evaluation showed facial asymmetry with loss of support in the right cheek and preservation of the orbital floor, with no evidence of enophthalmos (Figure 4). Maximum mouth opening was limited, and the patient's speech was difficult to understand.

The limitations imposed by trismus, combined with the extent of the defect, were discussed for all stages of rehabilitation. An efficient closed hollow obturator, designed to seal the defect and incorporate dentition, was initially proposed. However, due to the intraoral insertion and removal constraints, a two-piece closed hollow obturator retained by magnetic attachment was considered a viable alternative.

Five appointments were planned with the following respective goals: preliminary impressions, definitive impression, jaw relation record, teeth try-in, and insertion. Postinsertion denture adjustment appointments would be scheduled as needed.

**Figure 5:**
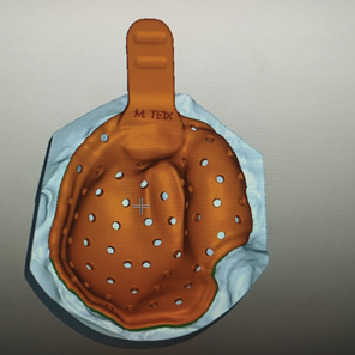
CAD of the custom impression tray.

In the first appointment, bypassing the difficulties of performing an impression using a stock impression tray, the previous obturator was poured with gypsum type III (Pro-Solid Super, Pro-Dental, Germany) to produce a study cast. After the gypsum set, the obturator was recovered and delivered to the patient. The mandibular impression was taken using irreversible hydrocolloid (Tropicalgin, Zhermack) and stock impression trays (Doric Master Trays, Schottlander, United Kingdom). The preliminary casts were scanned (S 600, Zirkonzahn, Italy), and an upper custom tray was designed (Zirkonzahn Tray, Zirkonzahn, Italy) and 3D-printed (NextDent 5100, 3D Systems, the Netherlands) in light-polymerizing PMMA resin (NextDent Tray, 3D Systems, the Netherlands) (Figure 5).

**Figure 6:**
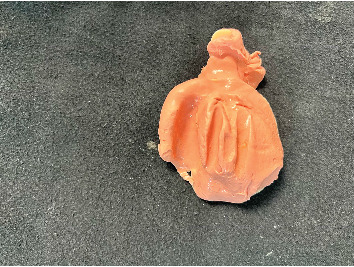
Alginate impression with custom tray.

**Figure 7:**
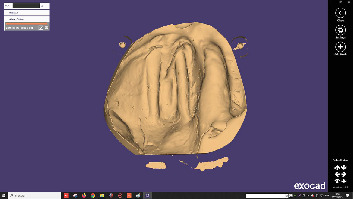
Final digital model.

**Figure 8:**
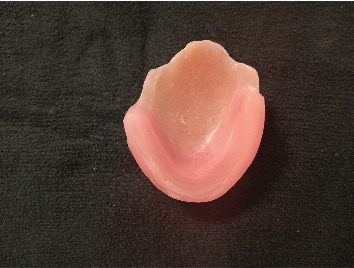
Registration plate with wax for bite registration.

In the second appointment, the upper final impression was made with irreversible hydrocolloid (Tropicalgin, Zhermack) (Figure 6). After boxing the impression, the master cast was obtained with gypsum Type III (Pro-Solid Super, Pro-Dental, Germany) and digitalized (S600, Zirkonzahn, Italy), providing the final digital model (Figure 7). Afterward, a conventional acrylic baseplate with wax occlusion rims was made (Figure 8).

**Figure 9:**
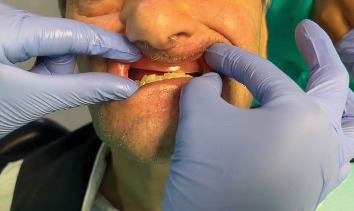
Intermaxillary registration stage, highlighting the difficulty in establishing the desired occlusal plane due to the limited mouth opening.

**Figure 10:**
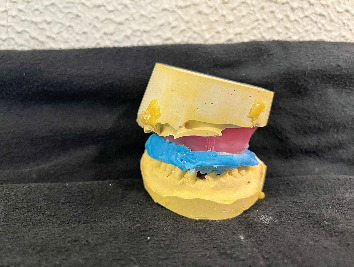
Maxillomandibular relationship record.

On the third appointment, the occlusal vertical dimension and jaw relation were recorded. There was a significant difficulty in inserting and removing the baseplate and occlusion rim due to the patient's trismus. Thus, the record had to be made using wax and silicone, allowing both parts to be removed separately (Figures 9 and 10). The jaw relation record was scanned using the Zirkonzahn scanner (S600, Zirkonzahn, Italy) to obtain digitally articulated jaws.

**Figure 11:**
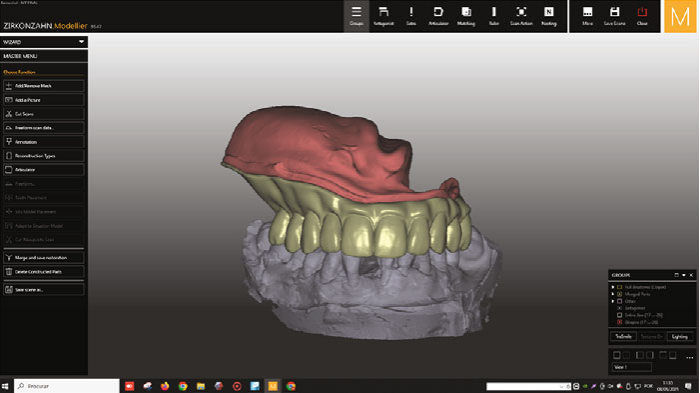
CAD of the two-pieces obturator (Zirkonzahn software).

**Figure 12:**
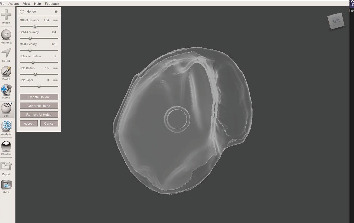
CAD design of the bulb part in Meshmixer software, illustrating the hollow section (dark gray) and the planned site for magnet placement.

Considering the difficulties encountered, a two-piece try-in was designed: a closed hollow bulb base component covering the entire defect and remaining edentulous ridge and a prosthetic segment containing the dental arch, intended to be placed over the bulb base. The teeth were selected from a digital library (AIDA, Zirkonzahn Library, Italy) and set using a modulation software program (Modellier by Zirkonzahn, Italy) (Figure 11). A space for magnet placement was planned between the two components to ensure their stable connection (Figure 12). Using a 3D printer (NextDent LCD1, NextDent B.V., the Netherlands), the two-piece obturator try-in was fabricated with a light-curing resin (NextDent Try-In, 3D Systems, the Netherlands). After printing, the try-in was ultrasonically cleaned in 99.5% isopropyl alcohol (AGA, Portugal) for 15 min and then placed in the LC-3D Print Box (NextDent B.V., the Netherlands) for final photopolymerization (10 min). Subsequently, two provisional magnets were positioned.

In the fourth appointment, the try-in was tested intraorally to assess insertion and removal feasibility, aesthetics, occlusion, and occlusal vertical dimension. Once all parameters were deemed satisfactory, the tooth shade was selected using a conventional shade guide (VITA Zahnfabrik), and the final two-piece obturator was fabricated. To obtain a closed hollow bulb, the STL file was exported to Autodesk Meshmixer software, and the hollow obturator part was designed using the ‘hollow' function in the Edit menu. The data for the entire obturator part were extracted using the ‘offset distance' tool, leaving a 1.5-mm thick outer border (Figure 12).

**Figure 13:**
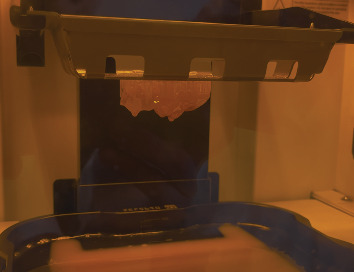
Printed hollow bulb (Part 1 of the obturator).

**Figure 14:**
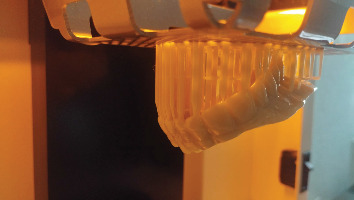
Printed prosthesis (Part 2 of the obturator).

**Figure 15:**
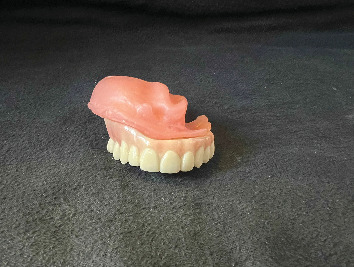
Characterized printed prosthesis (Part 2 of the obturator).

**Figure 16:**
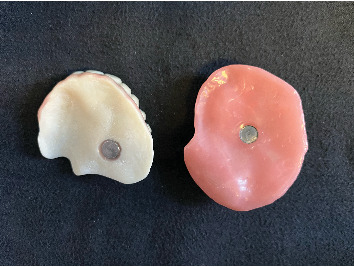
Magnetic interface between the two components of the obturator.

**Figure 17:**
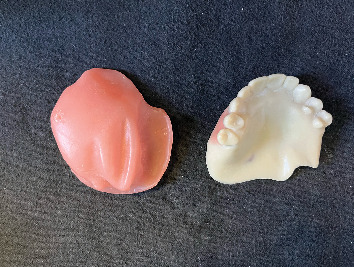
Occlusal and intaglio surfaces showing anterior retentive zones.

After this the bulb base was printed (NextDent LCD1, NextDent B.V., the Netherlands) using a pink resin (NextDent Base, 3D Systems, the Netherlands) (Figure 13), and the denture was printed using tooth resin color 1.5 (NextDent C&B MFH, 3D Systems, the Netherlands) (Figure 14). After printing, the dentures and bulb base were exposed to the previously explained final cleaning and photopolymerization procedures. Then, the denture's gingival area was covered with a thin layer of pink autopolymerizing acrylic (Probase Auto, Ivoclar Vivadent), and the two definitive magnets (Multipurpose Magnet, Technovent, United Kingdom) were fixed with pink resin (NextDent Base, 3D Systems, the Netherlands) (Figures 15, 16, and 17). Each tooth was also individually pigmented with a photopolymerized acrylic (VITA Akzent LC, VITA Zahnfabrik, Germany) for a more natural appearance.

**Figure 18:**
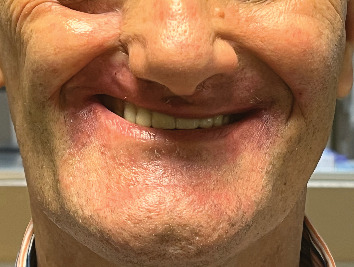
Frontal view of the inserted two-piece obturator.

In the fifth appointment, for denture insertion, the patient was instructed to place the bulb segment first, followed by the dental segment, ensuring that the magnets engaged properly. For the removal, the patient was advised to insert a finger into the interface between the two components in the left lateral vestibule and apply gentle pressure to separate them. Only minor adjustments to the base and occlusal surfaces were necessary (Figure 18).

At the 1-week follow-up, the patient reported regular use of the prosthesis and complained of pain in the edentulous crest. Upon clinical examination, the pressure area was identified, and appropriate relief was provided.

## 3. Discussion

The surgical resection of a malignant lesion involving the maxilla can result in substantial oromaxillary defects, significantly compromising the patient's functional capacity in daily activities. In such cases, the primary objective of prosthetic rehabilitation is to close the communication between the oral and nasal cavities using an obturator. Additionally, treatment aims to restore the oral cavity's aesthetics and function.

The fabrication of an obturator becomes particularly challenging in the presence of trismus associated with extensive anatomical defects. In this case, the registration step revealed the difficulty of inserting a single-piece prosthesis, which led to the decision to fabricate a two-piece obturator. Although a single-piece prosthesis is generally preferred by patients due to its simplicity and ease of handling, in cases of trismus combined with significant maxillary recession or large prosthetic dimensions, insertion is only feasible when the prosthesis is fabricated as two separate components joined by a magnetic retention system. Magnetic systems have been optimized, with improved corrosion resistance and magnetic force, thereby enhancing their reliability and effectiveness in dental applications [14, 17].

The presence of trismus also significantly limited the impression stage, making considerations necessary. Digital impression with intraoral scanner was not considered due to the inability to insert an intraoral scanner. Consequently, the old obturator was used as a reference for the first digital model. Instead of scanning the acrylic prosthesis directly, it was cast in gypsum and then digitized, as gypsum provides superior surface readability compared to shining acrylic surface. After that, a custom tray was designed and 3D-printed with perforations and relief areas, enabling the use of alginate as the impression material. Although silicone is generally preferred for definitive impressions, alginate was selected for two main reasons: its flexibility allowed easier removal in the presence of restricted mouth opening, and it minimized the risk of traumatizing the nasal mucosa, which was often affected by undercuts and small blood clots. Since alginate, a more viscous material than silicone, was chosen, border molding with modeling compound was not performed. Instead, only adjustments to the tray borders were made to avoid tissue compression.

In addition to the challenges associated with obturator insertion, the anatomical structures within the residual maxilla and the acquired defect must be carefully evaluated to ensure adequate prosthesis retention and stability. In this case, the remaining maxillary segment was edentulous, making it more difficult to achieve retention compared to a dentate patient.

Furthermore, as the extent of the surgical resection increases, so does the weight of the prosthesis, raising significant concerns regarding retention. With larger defects, the force of gravity can exceed the capacity of the remaining anatomical substructures—whether teeth or bone—to retain the prosthesis, ultimately resulting in its displacement. A commonly adopted solution to this problem is the fabrication of hollow obturator bulbs, which allow for adequate prosthesis dimensions while maintaining a weight within acceptable limits [12, 13]. The CAD-CAM technology applied in this clinical case has proven to be an effective option for fabricating closed hollow bulbs [18]. This digital approach allows for more precise control of wall thickness, ensures faster production, and reduces the need for postprocessing adjustments or repairs.

Even with a lightweight obturator, issues related to retention and stability remain common challenges in the prosthodontic rehabilitation of maxillectomy patients. These challenges are particularly pronounced in fully edentulous maxillae. In such cases, and when systemic health and bone availability allow, implants should be considered in the contralateral edentulous ridge to improve retention. To further enhance support and stability, implant placement in areas surrounding the defect—such as the residual zygomatic bone—may also be indicated [19, 20].

In the present case, the use of implants in the contralateral edentulous ridge was initially considered. However, this approach was deemed unfeasible due to the presence of trismus and the limited prosthetic space available for components connecting the implants to the prosthesis. In such situations, beyond achieving proper adaptation, the use of retentive areas in the nasal cavity can provide additional retention. In this case, the obturator was designed with two anterior protrusions that engaged retentive zones (Figure 17). Nevertheless, given its considerable volume and weight, the satisfactory retention of the definitive obturator was primarily attributable to the establishment of a stable occlusion, which had been lacking in the temporary prosthesis previously used by the patient.

A general assessment of the case shows that the workflow remains complex, as digitalization has not yet provided full simplification, which is frequently interpreted as a decrease in the number of clinical visits. However, even in conventional complete dentures, a fully digital workflow is still not feasible [21]. Functional border molding and the digital recording of the interocclusal relationship remain major challenges. In this case, trismus further compounded these difficulties. For instance, it was not possible to fabricate a custom tray with an incorporated wax rim to perform both the impression and the record in a single appointment. The greatest advantage of digital technology in this situation was therefore the reduction of laboratory time and, most importantly, the possibility of 3D-printing a closed hollow bulb, which proved more efficient and reliable compared to the time-sensitive techniques of the conventional method.

## 4. Conclusion

In cases where mouth opening is limited due to surgery and radiotherapy, the fabrication of an obturator prosthesis presents significant challenges. When the vertical height of the prosthesis exceeds the patient's maximum oral opening, a two-piece obturator retained by neodymium magnets offers an effective solution, allowing for easier insertion and removal by the patient.

## Data Availability

Data sharing is not applicable to this article as no datasets were generated or analyzed during the current study.

## References

[1] World Health Organization Ageing. https://www.who.int/health-topics/ageing#tab=tab_1.

[2] Farghal A. E. (2023). Fabrication of a Definitive Obturator for a Patient With a Maxillary Defect: A Case Report. *Cureus*.

[3] Pinheiro P. S., Tyczyński J. E., Bray F., Amado J., Matos E., Parkin D. M. (2003). Cancer Incidence and Mortality in Portugal. *European Journal of Cancer*.

[4] Howard A., Agrawal N., Gooi Z. (2021). Lip and Oral Cavity Squamous Cell Carcinoma. *Hematology/Oncology Clinics of North America*.

[5] Petti S., Scully C. (2005). Oral Cancer: The Association Between Nation-Based Alcohol-Drinking Profiles and Oral Cancer Mortality. *Oral Oncology*.

[6] Marur S., D'Souza G., Westra W. H., Forastiere A. A. (2010). HPV-Associated Head and Neck Cancer: A Virus-Related Cancer Epidemic. *Lancet Oncology*.

[7] Wertheimer-Hatch L., Hatch G. F., HatchB S. K. F. (2000). Tumors of the Oral Cavity and Pharynx. *World Journal of Surgery*.

[8] Sun K., Tan J. Y., Thomson P. J., Choi S. W. (2023). Influence of Time Between Surgery and Adjuvant Radiotherapy on Prognosis for Patients With Head and Neck Squamous Cell Carcinoma: A Systematic Review. *Head & Neck*.

[9] Likhterov I., Roche A. M., Urken M. L. (2019). Contemporary Osseous Reconstruction of the Mandible and the Maxilla. *Oral and Maxillofacial Surgery Clinics of North America*.

[10] Aramany M. A. (1978). Basic Principles of Obturator Design for Partially Edentulous Patients. Part I: Classification. *Journal of Prosthetic Dentistry*.

[11] Keyf F. (2001). Obturator Prostheses for Hemimaxillectomy Patients. *Journal of Oral Rehabilitation*.

[12] Spatz H. I., Schmitz J. T., Singh A. (2025). Comparison of the Weight of Conventionally Heat-Processed Hollow and Solid Obturators and 3D Printed Hollow Obturators. *Journal of Prosthetic Dentistry*.

[13] Alfaraj A., Su F. Y., Lin W. S. (2022). CAD-CAM Hollow Obturator Prosthesis: A Technical Report. *Journal of Prosthodontics*.

[14] Kapetanakos M., Golden M., Huryn J. M. (2020). Rehabilitation of a Patient After a Total Maxillectomy With a 2-Piece Magnetically Retained Obturator: A Clinical Report. *Journal of Prosthetic Dentistry*.

[15] van der Geer S. J., Kamstra J. I., Roodenburg J. L. (2016). Predictors for Trismus in Patients Receiving Radiotherapy. *Acta Oncologica*.

[16] Alqarni H., Alfaifi M., Ahmed W. M., Almutairi R., Kattadiyil M. T. (2023). Classification of Maxillectomy in Edentulous Arch Defects, Algorithm, Concept, and Proposal Classifications: A Review. *Cllinical and Experimental Dental Research*.

[17] Parameswari B. D., Rajakumar M., Jagadesaan N., Annapoorni H. (2017). Case Presentation of Two Maxillectomy Patients Restored With Two-Piece Hollow Bulb Obturator Retained Using Two Different Types of Magnets. *Journal of Pharmacy & Bioallied Sciences*.

[18] Gomes I., Martins J. P., Lopes L. P. (2024). Fabrication of a Closed Hollow Obturator by Digital Technologies: Technical Report. *Revista Portuguesa de Estomatologia, Medicina Dentaria e Cirurgia Maxilofacial*.

[19] Bidra A. S., Jacob R. F., Taylor T. D. (2012). Classification of Maxillectomy Defects: A Systematic Review and Criteria Necessary for a Universal Description. *Journal of Prosthetic Dentistry*.

[20] Noh K., Pae A., Lee J. W., Kwon Y. D. (2016). Fabricating a Tooth- and Implant-Supported Maxillary Obturator for a Patient After Maxillectomy With Computer-Guided Surgery and CAD/CAM Technology: A Clinical Report. *Journal of Prosthetic Dentistry*.

[21] Chebib N., Imamura Y., El Osta N., Srinivasan M., Müller F., Maniewicz S. (2024). Fit and Retention of Complete Denture Bases: Part II - Conventional Impressions Versus Digital Scans: A Clinical Controlled Crossover Study. *Journal of Prosthetic Dentistry*.

